# PReS-FINAL-2188: Insulin sensitivity is improved in sjia children with insulin resistance after tocilizumab treatment: results from the tender study

**DOI:** 10.1186/1546-0096-11-S2-O23

**Published:** 2013-12-05

**Authors:** H Mirjafari, N Ruperto, HI Brunner, Z Zuber, F Zulian, MR Maldonado-Velázquez, E Mantzourani, K Murray, J Roth, J Rovensky, O Vougiouka, J Wang, O Harari, D Lovell, A Martini, F De Benedetti

**Affiliations:** 1 Arthritis Research UK Epidemiology Unit, Institute of Inflammation and Repair, Manchester Academic Health Science Centre, The University of Manchester, Manchester, UK; 2 Roche, Welwyn, UK; 3 PRINTO, Genoa, Italy; 4 PRCSG, Cincinnati, USA; 5 IRCCS Ospedale Ped Bambino Gesu, Rome, Italy

## Introduction

In adults with inflammatory arthritis, insulin resistance (IR) is associated with diabetes and cardiovascular disease. Interleukin-6 (IL-6) is postulated to play a mechanistic role in IR.

## Objectives

To evaluate the degree of IR among children with systemic juvenile idiopathic arthritis (sJIA) and whether treatment with tocilizumab (TCZ) results in attenuation of IR in sJIA.

## Methods

Patients (pts) from TENDER^1 ^were included if baseline and wk 6 fasting insulin were measured. Glucocorticoid tapering was not permitted until wk 6. Insulin sensitivity was quantified using the homeostatic model of insulin resistance (HOMA-IR). Pts were classified as having IR if their HOMA-IR was ≥2.2 U. Change in HOMA-IR after 6 wks was assessed using paired *t*-test. Baseline associations with HOMA-IR and factors predicting change of HOMA-IR from baseline were assessed using regression analyses. Factors changing in association with HOMA-IR change were assessed.

## Results

92 pts with sJIA were analysed. 62 were randomised to TCZ and 30 to placebo, 12 of whom required escape therapy with TCZ by wk 6. At baseline, 40 pts (43%) had IR. Baseline HOMA-IR was associated with higher standardised body mass index and higher IL-6 levels (β-coefficient [95% CI]: 0.20 [0.05, 0.35] and 0.019 [0.001, 0.038], respectively) but not with JADAS, CRP, active joint count or presence of fever. Of the 74 pts who received TCZ, 34 (46%) had IR at baseline, including 4 pts who escaped from the placebo arm, compared with 6/18 (33%) who received only placebo. IR pts treated with TCZ but not placebo had significant reductions in HOMA-IR at wk 6 (Table). Across all IR pts, improvement in JADAS and active joint count was not associated with improvement in HOMA-IR (β-coefficient [95% CI]: 0.04 [-0.07, 0.14] and 0.08 [-0.06, 0.22], respectively).

**Table 1:** Change in HOMA-IR after 6 wks of Treatment in Children with sJIA in the TENDER Study

	Change in HOMA-IR Mean (95% CI)	*p*-value*
**TCZ (n = 34)**	-0.2 (-3.8, -0.2)	0.03

**PBO (n = 6)**	0 (-1.7, 1.6)	NS

NS, not significant; PBO, placebo. *paired *t*-test.

## Conclusion

After only 6 wks of TCZ treatment, HOMA-IR was improved in IR pts with sJIA in the presence of unchanged glucocorticoid dose. These data support a mechanistic contribution of IL-6 to IR in vivo in humans.

## Disclosure of interest

H. Mirjafari Employee of: Roche, N. Ruperto Grant/Research Support from: Abbott, AstraZeneca, BMS, Centocor, Lilly, Francesco Angelini, GSK, Italfarmaco, Merck Serono, Novartis, Pfizer, Regeneron, Roche, Sanofi Aventis, Schwarz Biosciences GmbH, Xoma, Wyeth, H. Brunner Consultant for: Novartis, Genentech, MedImmune, EMD Serono, AMS, Pfizer, UCB, Jannsen, Speakers Bureau: Genentech, Z. Zuber: None Declared, F. Zulian: None Declared, M. R. Maldonado-Velázquez: None Declared, E. Mantzourani: None Declared, K. Murray: None Declared, J. Roth: None Declared, J. Rovensky: None Declared, O. Vougiouka: None Declared, J. Wang Employee of: Roche, O. Harari Shareholder of: Roche, Employee of: Roche, D. Lovell Grant/Research Support from: NIH, Consultant for: AstraZeneca, Centocor, Janssen, Wyeth, Amgen, BMS, Abbott, Pfizer, Regeneron, Hoffmann-La Roche, Novartis, Genentech, Speakers Bureau: Genentech, Roche, A. Martini Grant/Research Support from: Abbott, AstraZeneca, BMS, Janssen, Lilly, Francesco Angelini, GSK, Italfarmaco, Novartis, Pfizer, Roche, Sanofi Aventis, Schwarz Biosciences GmbH, Xoma, Wyeth, The Gaslini Hospital, Consultant for: Abbott, AstraZeneca, BMS, Janssen, Lilly, Francesco Angelini, GSK, Italfarmaco, Novartis, Pfizer, Roche, Sanofi Aventis, Schwarz Biosciences GmbH, Xoma, Wyeth, The Gaslini Hospital, Speakers Bureau: Astellas, AstraZeneca, BMS, GSK, Italfarmaco, MedImmune, Novartis, F. De Benedetti Grant/Research Support from: Abbott, Pfizer, BMS, Roche, Novimmune, Novartis, SOBI.
